# Seroprevalence of *Bartonella henselae* and *Bartonella quintana* Infection and Impact of Related Risk Factors in People from Eastern Slovakia

**DOI:** 10.3390/pathogens10101261

**Published:** 2021-09-29

**Authors:** Katarína Petríková, Monika Halánová, Ingrid Babinská, Mariia Logoida, Karin Kaliariková, Peter Jarčuška, Sylvia Dražilová, Vladimíra Sobolová, Martin Janičko

**Affiliations:** 1 Department of Epidemiology, Faculty of Medicine, Pavol Jozef Šafárik University in Košice, 040 11 Košice, Slovakia; katarina.petrikova@student.upjs.sk (K.P.); ingrid.babinska@upjs.sk (I.B.); mariia.logoida@student.upjs.sk (M.L.); karin.kaliarikova@gmail.com (K.K.); 2 2nd Department of Internal Medicine, Faculty of Medicine, Pavol Jozef Šafárik University, 040 11 Košice, Slovakia; peter.jarcuska@upjs.sk (P.J.); sylvia.drazilova@upjs.sk (S.D.); martin.janicko@upjs.sk (M.J.); 3 Department of Urology, Faculty of Medicine, Pavol Jozef Šafárik University, 040 11 Košice, Slovakia; vladimira.sobolova@student.upjs.sk

**Keywords:** *Bartonella henselae*, *Bartonella quintana*, IgG antibodies, seroprevalence, risk factors, Slovakia

## Abstract

The genus *Bartonella* is a rapidly expanding group of ubiquitous bacteria that occur mainly in different animal species, but some can also be transmitted to humans. Three species, *B. henselae*, *B. bacilliformis*, and *B. quintana*, are responsible for the majority of human cases. The severity of the clinical symptoms often depends on the immune status of the patient, but others factors such as the species of the pathogen, virulence factors, and bacterial load also can play an important role. As the information on the occurrence of bartonellosis in the human population in Slovakia is absent, the aim of our pilot study was to determine the seroprevalence against *B. henselae* and *B. quintana* in the population of people living in Eastern Slovakia, and to identify the impact of related risk factors. Of 536 people included in the study, 126 (23.5%) showed positivity for anti-*B. henselae* antibodies and 133 (24.8%) against *B. quintana*. A statistically higher prevalence was confirmed only in the case of *B. quintana* in women regardless of the risk group. In analyzing the risk factors, we found significant differences between *B. henselae* seropositive and seronegative groups only in uric acid levels and serum creatinine, both, however, clinically irrelevant. Significant, but clinically irrelevant differences were observed also in alanine aminotransferase (ALT) levels and creatinine in people seropositive to *B. quintana*.

## 1. Introduction

*Bartonella* infections, belonging to a group of emerging diseases, are caused by a Gram-negative, aerobic, pleomorphic, intracellular pathogens of the order Rhizobiales, genus *Bartonella*.

Bartonellosis was first described in 1909, and by 1990 only two species of *Bartonella* were known. Since the last reclassification in 1993, the number of *Bartonella* species has increased to 45 [1].

Among all the *Bartonella* species, there are at least 16 species that have been associated with the human disease—*Bartonella* (*B.*) *alsatica*, *B. ancashensis*, *B. bacilliformis*, *B. bovis*, *B. clarridgeiae*, *B. doshiae*, *B. elizabethae*, *B. grahamii*, *B. henselae*, *B. koehlerae*, *B. mayotimonensis*, *B. quintana*, *B. rochalimae*, *B. tamiae*, *B. vinsonii* subsp. *arupensis* and *B. vinsonii* subsp. *berkhoffi* [1,2,3]. With the exception of *B. bacilliformis*, which has a human reservoir, other species are zoonotic. Multiple reservoirs for the development of human *Bartonella* infections are both domestic and wild animals. In Europe, these are mainly cats, dogs, and rodents [4,5].

Three species—*B. henselae*, *B. bacilliformis*, and *B. quintana*—are mainly responsible for most human *Bartonella* diseases.

*B. henselae* is the etiological agent of one of the most well-known zoonotic human infections caused by *Bartonella*—cat scratch disease (CSD) [6]. Carrion’s disease, endemic to South American Andean valleys, is caused by the *B. bacilliformis* [7], and *B. quintana* is the causative agent of Trench fever [8]. 

Diseases caused by these microorganisms are mainly transmitted by the bite or scratch of infected animals or by different insect vectors, while some arthropods were proved as a vector of *Bartonella* infections (e.g., fleas, flies, lice) and some are suspected to be involved in transmission (e.g., some species of bed bugs, ticks, red ants, spiders), depending on the bacteria species and their vertebrate reservoirs [1,2,9,10,11,12,13,14]. Increased interest in *Bartonella* became evident only in the early 1990s, when *B. quintana* and *B. henselae* were first described in HIV-infected patients and subsequently in immunocompetent persons [15]. Infections caused by these pathogens are responsible for a variety of manifestations from asymptomatic or mild symptoms, such as low-grade fever, malaise, regional lymphadenopathy, and headache, to more severe symptoms, such as endocarditis, meningitis, osteomyelitis, hemolytic anemia, hepatosplenomegaly, glomerulonephritis, and encephalitis [1,16]. The severity of clinical symptoms often depends on the immune status of the patient, but others factors such as the species of the pathogen, virulence factors, and bacterial load also can play an important role [3]. 

The prevalence of *Bartonella* spp. varies among different geographic regions, groups of examined people, and the diagnostic method used [17]. Most of the results were obtained through serological surveys to estimate the incidence of human bartonellosis [3]. 

As there is no law in Slovakia that imposes systematic screening for this infection, information on the occurrence of this disease in humans are absent. Thus far, this infection has been confirmed in Slovakia only in small mammals, with positivity ranging between 9% and 73.8% [18,19]. 

Risk factors associated with *Bartonella* infections include direct contact with infected animals (especially with stray cats and rodents). A variety of insects, including fleas, body lice, and ticks, act as vectors and play an important role in transmission not only between animals but also between animals and humans [2,10]. Their occurrence is most commonly associated with homelessness or areas of high population density and poor sanitation [20]. For these reasons, unsatisfactory social conditions and insufficient medical care are also described as potential risk factors. Therefore, the aim of our study was to determine the prevalence of antibodies against *B. henselae* and *B. quintana* in the population of people living in Eastern Slovakia and to identify the impact of related risk factors.

## 2. Results

### 2.1. Baseline

Analysis of baseline information of the people living in segregated Roma settlements (risk group) and people from the general population showed that this two populations are completely different in terms of demographics, socioeconomic status, and biochemical parameters (except transaminases), (Table 1).

### 2.2. Antibodies Prevalence

In the case of a positive immunological response to the presence IgG antibodies against *B. henselae* and *B. quintana*, an apple-green fluorescence was detected. Samples that showed fluorescence at titers of 1:64 and above were considered positive.

Of the 536 respondents included in the study, 126 (overall prevalence 23.5%, 95% CI 20.0–27.1) showed positivity for IgG antibodies against *B. henselae*. In the case of *B. quintana*, IgG antibodies were confirmed in 133 people and overall prevalence was 24.8% (95% CI 21.3–28.7). Basic differentiation of seroprevalence based on gender and risk group is presented in Table 2. We observed no statistically significant difference in any antibody prevalence between Roma (risk group) and non-Roma participants. Irrespective of risk group, women had statistically higher prevalence of *B. quintana* IgG positivity and no difference was observed in *B. henselae* IgG seropositivity in regards to gender or risk group.

### 2.3. Risk Factors for Antibodies Prevalence

Because of no significant difference in any antibody prevalence between Roma and non-Roma, differences in potential risk factors were analyzed in the whole cohort (Table 3).

Roma ethnicity was entered as a confounding factor, along with gender in the multivariate regression model (Table 4). After adjustment for ethnicity and gender only, serum creatinine was marginally inversely associated with *B. henselae* seropositivity, but with low OR. Analogous analysis with *B. quintana* showed strong association with female gender (OR 2.1) and weaker association with basic education. All other analyzed risk factors were insignificant.

## 3. Discussion

Several new species and subspecies of the genus *Bartonella* have been characterized since the beginning of the 1990s. As a result, the spectrum of natural reservoirs, vectors, and subsequent human diseases caused by these species has expanded considerably [17].

In our study, we focused on the investigation of the prevalence of anti-*B. henselae* and anti-*B. quintana* antibodies in people living in Eastern Slovakia and identification of risk factors that may be involved in the development of the disease.

In the case of *B. henselae*, the most prevalent zoonotic *Bartonella* species occurring endemically worldwide, cats are the primary reservoir, but DNA from this pathogen has also been found in dogs, cows, horses, marine mammals, and sea turtles [3,21,22,23]. Infected cats usually have asymptomatic bacteremia lasting several months [3]. Transmission to humans occurs directly through a skin injury after a cat scratching or biting and may occur after being bitten by cat fleas (*Ctenocephalides felis*) also [3,24]. Other vectors, such as ticks, mites, and spiders are also an equivalent vectors for *B. henselae* and can play an important role in their transmission to humans and animals [3,25].

*B. quintana* has a worldwide distribution often associated with war zones and poor hygiene, which is related to the predisposition to contamination of the human body louse (*Pediculus humanus humanus*) [26,27]. This bacteria is an etiological agent of Trench fever and is also one of the pathogens of bacillary angiomatosis occurring in immunodeficient patients [28]. In addition, it may be involved in endocarditis and chronic bacteremia in people living in poor conditions (e.g., in homeless people), and is referred to as “urban trench fever” [29]. DNA of *B. quintana* has been isolated from feline fleas and ticks, and this bacteria has been confirmed in dogs, in addition to feral cats [30,31,32].

Generally, *Bartonella* infection in humans can be difficult to diagnose. Culture, molecular, and serological tests can be used, but since the causative agent cannot be easily cultured, the diagnosis usually relies on epidemiological, clinical, and serological proof.

In Slovakia, to date, bartonellosis in humans has not been officially reported. As is known from foreign studies, the occurrence of many *Bartonella* species is often associated with poor hygiene, unfavorable social conditions, and a low standard of living. In Slovakia, these conditions mainly concern people living in segregated Roma settlements. There is generally a higher prevalence of infectious diseases, poisoning, and injuries in this group [33]. These places are also characterized by a huge number of stray animals (especially dogs, cats, and rodents), which may have a direct share in their incidence.

Although our study confirmed that Roma live in socially weaker conditions compared to the majority (Table 1), and it could be expected that the incidence of *Bartonella* infections would be higher in the Roma population due to poor hygienic standards and increased contact with stray animals as a risk factor, our study did not confirm this. A statistically higher prevalence was confirmed only in the case of *B. quintana* in women regardless of the risk group. The explanation might be that having high hygienic standards in an area where other community could not afford it and where stray animals (especially stray cats) are increasingly present without proper management of risk does not prevent *Bartonella* infections efficiently.

In analyzing the risk factors, we found significant differences between *B. henselae* seropositive and seronegative groups only in uric acid levels and serum creatinine, both, however, clinically irrelevant. Significant, but clinically irrelevant differences were observed also in ALT levels and creatinine in people seropositive to *B. quintana*.

The overall seropositivity (23.5% for *B. henselae* and 24.8% for *B. quintana*) we found is comparable to the results of other studies. Generally, the prevalence of *Bartonella* infections in humans varies from 0.2% to 30% on average, depending on the investigated group and the diagnostic method used. For example, in a study conducted in Germany, the seropositivity rates among the healthy adults are 30%, in Greece 19.8%, in Poland 25.85%, in Austria 26.5%, in Sweden 16%, and 8.7% in Spain [25,34,35,36]. On the other hand, bartonellosis in Slovakia is not subject to mandatory reporting and, therefore, it is not possible to accurately determine incidence and prevalence. Because of the different results between studies and the overall lack of microbiological data in clinical and therapeutic studies, many problems have been encountered in diagnosis and subsequent therapy not only in humans but also in infected animals. Therefore, there is a need to further investigate *Bartonella* infections in the population and to study associations of the occurrence of *Bartonella* antibodies in humans and animals.

However, our study also has some limitations. First, the respondents were not asked directly about arthropod exposure or animal scratch, and we only hypothesize that it might happen, as it is quite common. However, the relevant exposure period is quite long and many respondents might not remember the accident, so it is questionable how reliable the retrospective data on it would be.

Another limitation may be the diagnostic test used. In our study, detection of IgG antibodies to *Bartonella* infections was done using a commercial indirect immunofluorescent assay (IFA) kit. This test uses Vero cells that have been infected with either *B. henselae* or *B. quintana* as the individual substrates permitting the qualitative detection and semi-quantitation of human serum IgG antibodies to *Bartonella*. This test uses only a small blood sample for reliable diagnosis; however, cross-reactive between species may occur and, therefore, the results should be interpreted with caution.

## 4. Materials and Methods

### 4.1. Study Population

Samples and data from the cross-sectional population-based Hepa-Meta study conducted in Eastern Slovakia were used. The study focused on the prevalence of viral hepatitis diseases, metabolic syndrome, and selected bacterial and parasitic infectious diseases in the population living in segregated settlements. The inhabitants from the general population from the same region were used as a control group. The methodology of this study was previously described in detail by Madarasová Gecková et al. [37].

The final sample that examined the presence of IgG antibodies against *B. henselae* and *B. quintana* was comprised of 536 people aged 18–55, randomly selected from the overall HepaMeta database (n = 855). The study cohort consisted of 283 people living in 10 different segregated Roma settlements in Eastern Slovakia (mean age = 35.08; SD = 9.28; 32.9% men) and 253 people from the general population from the same region (mean age = 32.61; SD = 7.23; 47% men) who were used as a control group.

Data were collected via analyzed blood and a questionnaire. Venous blood was collected from *vena mediana cubiti* under standard conditions. After centrifugation of the blood, serum samples were collected and stored at −80 °C until their use in the immunofluorescence test. Hyperlipemic, hemolyzed, and contaminated sera were excluded from testing.

The questionnaire gathered information about the sociodemographic background (employment status, education), living conditions (type of house, the number of people per house, availability of in-house sanitary equipment, electricity supply), and poverty (aggregate of the issue to pay at least one item of the following: rent, loan payment, healthcare, energies, other expenses).

### 4.2. Laboratory Examination

Detection of IgG antibodies to *Bartonella* infections was done using a commercial indirect immunofluorescent assay (IFA) kit (Focus Diagnostics, Cypress, CA, USA).

A kit that uses Vero cells infected with either *B. henselae* or *B. quintana* for detecting IgG antibodies was used according to the manufacturer’s instructions.

Antibody determination was performed in two phases. In the first step, the blood sera were diluted at a 1:64 screening dilution and then added to appropriate slide wells containing *B. henselae* and *B. quintana* antigens. After incubation for 60 min at room temperature and then washing, the fluorescently labelled anti-IgG antibodies were added to the wells. Afterwards, the slide was washed, dried and mounted and was examined using fluorescence microscopy at 400× magnification.

Biochemical parameters—aspartate aminotransferase (AST), alanine aminotransferase (ALT), uric acid, albumin, creatinine, and C-reactive protein (CRP) were determined by routine biochemical methods on the analyzer ADVIA 2400. Reference values for men were for AST 0.17–0.85 ukal/L, ALT 0.3–0.8 ukat/L, uric acid 220–420 ukat/L, albumin 32–48 g/L, creatinine 66–106 umol/L, and CRP 0–5 mg/L. For women the reference values were for AST 0.17–0.6 ukal/L, ALT 0.2-0.7 ukat/L, uric acid 140–340 ukat/L, albumin 32–48 g/L, creatinine 44–84 umol/L, and CRP 0–5 mg/L.

### 4.3. Statistical Analyses

Categorical variables are reported as absolute and relative frequency (n (%)). Interval variables are reported as mean ± standard error of mean. Baseline comparisons of the prevalence between groups based on various demographic, socio-economic, and laboratory variables were performed by Chi-squared test in case of categorical variables or Mann–Whitney test in case of interval variables. Adjusted odds ratios of variables that significantly differed between positive and negative groups were obtained by multivariate logistic regression adjusted for confounders. Two-sided *p* value < 0.05 was considered statistically significant.

## Figures and Tables

**Table 1:** Baseline differences between Roma (n = 283) and non-Roma (n = 253) population in Eastern Slovakia.

Parameter	Roma n (%)	Non-Roma n (%)	*p* Value
**demographic variables**			
Age, mean (STD)	35.1 (0.6)	32.6 (0.5)	0.001
Male gender	93 (32.9)	119 (47)	0.001
**socioeconomic variables**			
Basic education	228 (82.6)	3 (1.2)	<0.0001
Intermediate education	48 (17.4)	150 (61)	
Higher education	0 (0)	93 (37.8)	
Unemployment	250 (90.9)	62 (25.7)	<0.0001
Poverty	134 (47.3)	20 (7.9)	<0.0001
Bricked house	265 (95.3)	231 (100)	<0.0001
Missing household equipment	188 (66.4)	45 (17.8)	<0.0001
Number of persons in house, mean (STD)	7.5 (0.2)	4.3 (0.3)	<0.0001
No preventive medical check-up	146 (52.5)	82 (33.7)	<0.0001
**laboratory variables**			
AST (ukat/L)	0.31 ± 0.03	0.32 ± 0.01	0.673
ALT (ukat/L)	0.24 ± 0.24	0.25 ± 0.01	0.629
Uric Acid (umol/L)	223.3 ± 4.9	253.8 ± 5.6	<0.0001
Albumin (g/L)	46.6 ± 0.2	47.2 ± 0.2	0.026
Creatinine (umol/L)	82.1 ± 0.7	85.4 ± 0.8	0.001
CRP (mg/L)	3.1 ± 0.24	1.99 ± 0.18	<0.0001

**Table 2:** Prevalence of anti-*B. henselae* and anti-*B. quintana* IgG based on risk group and gender.

	Roma		Non-Roma		*p* Value
	Male n (%)	Female n (%)	Male n (%)	Female n (%)	
***B. henselae* IgG**	21 (22.6)	46 (24.2)	20 (16.8)	39 (29.1)	0.145
***B. quintana* IgG**	21(22.6)	59 (31.1)	14 (11.8)	39 (29.1)	0.001

**Table 3:** Differences in potential risk factors between seropositive and seronegative patients.

Parameter	Anti-*B. henselae* IgG		*p* Value	Anti-*B. quintana* IgG		*p* Value
	Positive n (%)	Negative n (%)		Positive n (%)	Negative n (%)	
**Demographic variables**						
Age, mean (STD)	34.5 ± 7	33.8 ± 0.4	0.418	34.7 ± 0.8	33.7 ± 0.4	0.200
Male gender	41 (32.5)	171 (41.7)	0.076	35 (26.3)	177 (43.9)	<0.0001
**Socioeconomic variables**						
Basic education	54 (44.3)	177 (44.3)	0.997	72 (55.4)	159 (40.6)	0.012
Intermediate education	46 (37.7)	152 (38.0)		41 (31.5)	157 (40.1)	
Higher education	22 (18)	71 (17.8)		17 (13.1)	76 (19.4)	
Unemployment	71 (57.7)	241 (61.3)	0.526	85 (64.9)	227 (59)	0.256
Poverty	43 (34.1)	111 (27.1)	0.143	40 (30.1)	114 (28.3)	0.740
Bricked house	115 (98.3)	381 (97.2)	0.742	125 (98.4)	371 (97.1)	0.533
Missing household equipment	58 (46)	175 (42.7)	0.538	65 (48.9)	168 (41.7)	0.159
Number of persons in house, mean (STD)	5.9 ± 0.3	6.2 ± 0.25	0.517	6.42 ± 0.3	6.04 ± 0.3	0.421
No preventive medical check-up	51 (42.1)	177 (44.3)	0.754	51 (38.9)	177 (45.4)	0.222
**Laboratory variables**						
AST (ukat/L)	0.28 ± 0.01	0.33 ± 0.02	0.153	0.27 ± 0.01	0.33 ± 0.02	0.069
ALT (ukat/L)	0.21 ± 0.01	0.26 ± 0.02	0.115	0.19 ± 0.01	0.26 ± 0.02	0.002
Uric Acid (umol/L)	223.23 ± 7.5	242.25 ± 4.3	0.028	227.01 ± 6.8	241.32 ± 4.4	0.078
Albumin (g/L)	46.62 ± 0.3	46.97 ± 0.1	0.254	46.62 ± 0.2	46.97 ± 0.2	0.230
Creatinine (umol/L)	81.05 ± 0.9	84.45 ± 0.6	0.002	81.25 ± 0.8	84.45 ± 0.6	0.002
CRP (mg/L)	2.42 ± 0.3	2.64 ± 0.18	0.556	2.70 ± 0.37	2.55 ± 0.17	0.698

**Table 4:** Adjusted risk for *B. henselae* and *B. quintana* seropositivity.

Parameter	OR for Anti-*B. henselae* IgG Positivity	*p* Value	OR for Anti-*B. quintana* IgG Positivity	*p* Value
**demographic variables**				
Age	1.009 (0.984–1.033)	0.712	1.009 (0.986–1.034)	0.442
Male gender	0.671 (0.438–1.027)	0.066	0.476 (0.307–0.736)	0.001
Roma ethnicity	1.036 (0.691–1.554)	0.863	0.738 (0.492–1.107)	0.142
**socioeconomic variables**				
Basic education	0.984 (0.385–2.512)	0.972	3.031 (1.092–8.413)	0.033
Intermediate education	0.988 (0.537–1.816)	0.968	1.297 (0.674–2.495)	0.437
Higher education	Reference		Reference	
Unemployment	0.743 (0.422–1.307)	0.302	0.845 (0.477–1.496)	0.563
Poverty	1.53 (0.944–2.481)	0.084	0.922 (0.572–1.485)	0.738
Bricked house	1.922 (0.413–8.944)	0.405	2.751 (0.592–12.781)	0.197
Missing household equipment	1.157 (0.727–1.842)	0.539	1.090 (0.690–1.724)	0.712
Number of persons in house	0.981 (0.927–1.038)	0.507	1.0 (0.951–1.052)	0.998
No preventive medical check-up	0.907 (0.596–1.381)	0.651	0.680 (0.447–1.034)	0.072
**laboratory variables**				
AST	0.377 (0.066–2.150)	0.272	0.276 (0.035–2.193)	0.223
ALT	0.443 (0.121–1.623)	0.219	0.362 (0.086–1.517)	0.165
Uric Acid	0.998 (0.995–1.001)	0.124	1 (0.997–1.003)	0.894
Albumin	0.974 (0.907–1.046)	0.474	0.992 (0.926–1.063)	0.815
Creatinine	0.971 (0.947–0.996)	0.021	0.993 (0.970–1.017)	0.566
CRP	0.981 (0.925–1.041)	0.537	1.003 (0.950–1.059)	0.916

## Data Availability

The data presented in this study are available on request from the corresponding author.
